# Strength increase during ceramic biomaterial-induced bone regeneration: a micromechanical study

**DOI:** 10.1007/s10704-016-0157-z

**Published:** 2016-10-20

**Authors:** Stefan Scheiner, Vladimir S. Komlev, Christian Hellmich

**Affiliations:** 1 Institute for Mechanics of Materials and Structures, Vienna University of Technology, Vienna, Austria; 2 A.A. Baikov Institute of Metallurgy and Materials Science, Russian Academy of Sciences, Moscow, Russia; 3 Institute of Laser and Information Technologies, Russian Academy of Sciences, Moscow, Russia

**Keywords:** Continuum micromechanics, Elastic limit, Multiscale modeling, Bone ingrowth, Tissue engineering

## Abstract

Bone tissue engineering materials must blend in the targeted physiological environment, in terms of both the materials’ biocompatibility and mechanical properties. As for the latter, a well-adjusted stiffness ensures that the biomaterial’s deformation behavior fits well to the deformation behavior of the surrounding biological tissue, whereas an appropriate strength provides sufficient load-carrying capacity of the biomaterial. Here, a mathematical modeling approach for estimating the macroscopic load that initiates failure of a hierarchically organized, granular, hydroxyapatite-based biomaterial is presented. For this purpose, a micromechanics model is developed for downscaling macroscopically prescribed stress (or strain) states to the level of the needle-shaped hydroxyapatite crystals. Presuming that the biomaterial fails due to the quasi-brittle failure of the most unfavorably stressed hydroxyapatite needle, the downscaled stress tensors are fed into a suitable, Mohr-Coulomb-type failure criterion, based on which the macroscopic failure load is deduced. The change of the biomaterial’s composition in response to placing it in physiological solution, caused by growth of new bone tissue on the granules’s surfaces, on the one hand, and by resorption of the hydroxyapatite crystals, on the other hand, is taken into account by means of suitable evolution laws. Numerical studies show how the macroscopic load-carrying capacity of the biomaterial is influenced by its design parameters. The presented modeling approach could prove beneficial for the design process of the studied biomaterials (as well as similarly composed biomaterials), particularly in terms of optimizing its mechanical performance.

## Introduction

The field of bone tissue engineering aims at the reinforcing or even replacing diseased (or for other reasons malfunctioning) bone tissue by scaffold structures that are specifically engineered, for blending in the targeted physiological environment, i.e. the immediate vicinity of bone tissue, as well as possible (Burg et al. 2000; Reichert and Hutmacher 2011). From a mechanical point of view, careful tuning of such scaffold structures (and of the materials which they are made of) is called for because contradictory requirements must be brought in line—scaffold structures must be stiff enough to sustain all relevant mechanical load cases, but also soft enough to facilitate, through mechanobiological couplings (Klein-Nulend et al. 2005; Porter et al. 2009; Velasco et al. 2015), the integration into their bony environment. In this regard, two mechanical properties are of particular interest, both on material and structural levels: the stiffness, governing the elastic deformation behavior and therefore the forces attracted by the involved macro- and microstructures; as well as the strength, indicating the stress level that induces material failure.

In the present paper, we study one specific scaffold material that has been developed as bone replacement material with the human mandible as targeted application area (Komlev et al. 2002, 2003). This biomaterial is produced in form of porous, pre-cracked granules, composed of hydroxyapatite as main constituent, but also including various kinds of pore spaces of distinctively different characteristic lengths. After exposing this biomaterial to the targeted physiological environment prevailing in the immediate vicinity of mandibular bone tissue, two mechanisms are triggered, causing a progressing change of the material’s composition over time. On the one hand, bone tissue grows on the granule surfaces, while, on the other hand, concurrently the hydroxyapatite crystals are resorbed—in the long run, the scaffold material merges with the surrounding bone tissue.

In a first approach to analyzing their mechanical behavior, these granules underwent micro-computed tomography ($\mu$CT), and the resulting scans served as basis for combined Finite Element/micromechanics-based simulations (Dejaco et al. 2012, 2016). Here, as a (computationally more efficient) complement, we present a three-step, fully continuum micromechanics-based macro-to-meso-to-micro (stress and strain) downscaling scheme, linking in the end the quasi-brittle failure of single micrometer- or sub-micrometer-sized hydroxyapatite crystal needles to the overall strength of both millimeter-sized biomaterial scaffolds and composites comprising biomaterial scaffold and bone tissue, respectively. For this purpose, a number of homogenization concepts are adapted, extended, and combined, considering the pioneering contributions of Eshelby (1957), Hill (1963, 1965), Laws (1977, 1985), Hervé and Zaoui (1993); and also considering more recent contributions of Deudé et al. (2002), Dormieux et al. (2004), Fritsch et al. (2006), Bertrand and Hellmich (2009). Following Fritsch et al. (2009a, b), we feed the stress of the most unfavorably loaded hydroxyapatite needle into a suitable, Mohr-Coulomb-type failure criterion, and deduce then therefrom the corresponding ultimate macroscopic load bearable by the aforementioned granular, hydroxyapatite-based biomaterial (optionally containing ingrown bone tissue).

After introducing the fundamental modeling concept, together with the chosen model representation of the studied biomaterial, see Sect. 2, a mathematical model for downscaling of the mechanical loading, from the macroscopic to the hydroxyapatite needle scale, is presented, see Sect. 3. Then, a suitable failure criterion is elaborated in Sect.  4.1, and numerical studies show how the macroscopic mechanical loading inducing single hydroxyapatite needle failure changes with varying biomaterial composition. In order to simulate bone regeneration (which occurs after having placed the biomaterial in the targeted physiological environment), involving bone growth and scaffold resorption, suitable evolution laws are introduced, and the effects of different material input parameters on the model-predicted development of the load-carrying capacity over time are studied, see Sect. 5. A brief discussion closes the paper, see Sect. 6.

## Material and methods

### Characterization of the multi-porous hydroxyapatite tissue engineering scaffold material

The biomaterial investigated in this paper is a granular scaffold material, with the granules composed of carbonate-containing hydroxyapatite, the chemical composition of which reads as Ca$_{10}$(PO$_4$)$_6$(OH)$_{1.9}$(CO$_3$)$_{0.05}$. This material is produced based on the effect of immiscible liquids (Komlev et al. 2002, 2003), giving access to granules with diameters ranging from 50 to 2000 $\upmu$m, with the technically relevant granule diameter being approximately 1800 $\upmu$m (Dejaco et al. 2012).

**Fig. 1:**
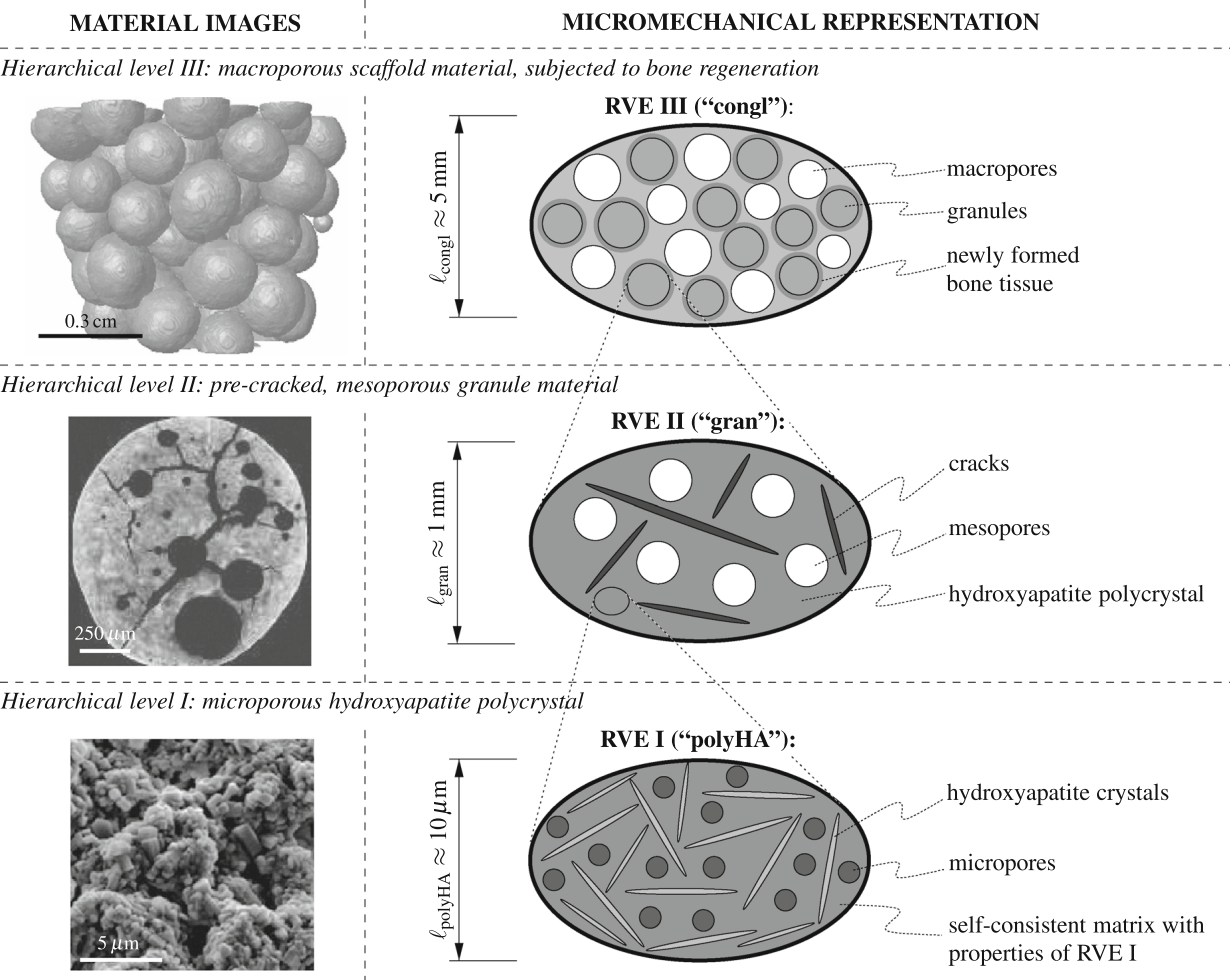
Three-level micromechanical representation of the hydroxyapatite-based granular biomaterial (*column* on the *right-hand side*), following the morphological features found in images on different observation scales (*column* on the *left-hand side*); the depicted images have been acquired by means of scanning electron microscopy (hierarchical level I) and $\mu$CT imaging techniques (hierarchical levels II and III)

Several morphological features of these granules can be observed, see the column on the left-hand side of Fig. 1. Firstly, the granules contain pores of two different characteristic lengths: small pores, with a characteristic length ranging from less than one to several micrometers (Dejaco et al. 2016)—these pores are termed “micropores” hereafter; and large pores, with a characteristic length of several hundred micrometers—these pores are termed “mesopores” hereafter. A composite of randomly oriented hydroxyapatite crystals and the micropores constitutes the “base material” of the granules. Increasing the observation scale by several orders of magnitude, one can discern, besides the mesopores, cracks pervading the granule body. Finally, the scaffold material is made up of the above described granules, with pore space in-between—due to the characteristic length of these pores, which is approximately equal to the granule diameter, they are termed “macropores” in the remainder of this paper.

### Fundamentals of continuum micromechanics: the representative volume element

A method particularly well suited for modeling the mechanical behavior of the material described in Sect. 2.1 is continuum micromechanics (Hill 1963; Zaoui 1997, 2002), where a material is understood as a micro-heterogeneous body filling a macro-homoge-neous representative volume element (RVE) with characteristic length $\ell$, $\ell \,\gg \,d$, *d* standing for the characteristic length of inhomogeneities within the RVE, and $\ell \,\ll \,{{\mathcal {L}}}$, ${{\mathcal {L}}}$ standing for the characteristic lengths of geometry or loading of a structure built up by the material defined on the RVE. It should be noted the aforementioned requirements of “much larger” ($\gg$) and “much smaller” ($\ll$), respectively, have been shown to be already satisfied if the respective characteristic lengths are separated by a factor of two to three and five to ten, respectively (Drugan and Willis 1996; Kohlhauser and Hellmich 2013).

In general, the microstructure within an RVE is too complicated to be described in complete detail. Therefore, quasi-homogeneous subdomains with known physical properties (such as volume fractions and mechanical properties) are reasonably chosen. They are called material phases. The homogenized (upscaled) behavior of the material on the observation scale of the RVE, i.e. the relation between homogeneous deformations acting on the boundary of the RVE and resulting macroscopic (average) stresses, can then be estimated from the mechanical behavior of the material phases, their volume fractions within the RVE, their characteristic shapes, and their interactions. If a single material phase is micro-heterogeneous itself, its mechanical behavior can be estimated by introduction of RVEs within this phase, with characteristic lengths $\ell _1\,\le \,d$, comprising again inhomo- geneities with characteristic length $d_1\,\ll \,\ell _1$, and so on. Such an approach is referred to as multi-step homogenization and provides, eventually, access to “universal” phase properties at sufficiently low observation scales (Fritsch and Hellmich 2007).

### Micromechanical modeling

Having in mind the concept of “separation of scales”, as introduced in Sect. 2.2, the following three-level micromechanical representation emerges for the biomaterial under investigation:

On *hierarchical level I*, a microporous, overall isotropic, hydroxyapatite polycrystal is composed of spherical micropores (with volume fraction $\phi _\text {micro}^\text {polyHA}$), which interact mutually with randomly oriented cylindrical hydroxyapatite crystals (with volume fraction $f_\text {HA}^\text {polyHA}=1-\phi _\text {micro}^\text {polyHA}$). Typically, the microporosity amounts to $\phi _\text {micro}^\text {polyHA}=0.445$ (Dejaco et al. 2012). The characteristic length of the polycrystalline RVE I is in the order of $10\,\upmu$m, see the bottom row of Fig. 1, with a scanning electron micrograph of the granule nano-structure on the left-hand side and the corresponding RVE I on the right-hand side. In terms of stiffness upscaling, the mutual mechanical interaction of all phases within RVE I calls for a self-consistent homogenization scheme, as introduced by (Fritsch et al. 2006), giving access to the stiffness tensor of the microporous hydroxyapatite polycrystal, $\mathbb {C}_\text {polyHA}$, based on the composition and morphology of RVE I, as well as on the stiffness tensors of the hydroxyapatite crystals, $\mathbb {C}_\text {HA}$, and of the micropores, $\mathbb {C}_{\text {micro}\phi }$.

On *hierarchical level II*, penny-shaped cracks (with vanishing volume fraction) and spherical mesopores (with volume fraction $\phi _\text {meso}^\text {gran}$) are embedded in the polycrystal matrix with properties arising from the structure of RVE I, this matrix filling within RVE II the volume fraction $f_\text {polyHA}^\text {gran}=1-\phi _\text {meso}^\text {gran}$. Typically, the mesoporosity comes to $\phi _\text {meso}^\text {gran}=0.189$ (Dejaco et al. 2012). The characteristic length of RVE II is in the order of 1 mm, see the middle row in Fig. 1, with a micro-computed tomography ($\mu$CT) image of the microstructure within a granule on the left-hand side and the corresponding RVE II on the the right-hand side. The distinctive matrix-inclusion morphology of RVE II—i.e. cracks and mesopores can be considered as inclusions embedded in the hydroxyapatite polycrystal matrix—suggests the use of a Mori-Tanaka-type homogenization scheme (Mori and Tanaka 1973; Benveniste 1987) for stiffness homogenization; mathematical treatment of the penny-shaped cracks has been dealt with by Deudé et al. (2002), Dormieux et al. (2004). The stiffness tensor of the pre-cracked, mesoporous granule material, $\mathbb {C}_\text {gran}$ is then governed by the composition and morphology of RVE II, as well as by the stiffness tensors of the hydroxyapatite polycrystal matrix, $\mathbb {C}_\text {polyHA}$, accessible from stiffness homogenization across RVE I, and of the mesopores, $\mathbb {C}_{\text {meso}\phi }$, and by the density of cracks, quantified by the so-called crack density parameter *e* (Budianksy and O’Connell 1976).

On *hierarchical level III*, a macroporous conglomerate material consisting of mesoporous, cracked hydroxapatite granules and newly grown bone tissue emerges, see the top of Fig. 1: granules with the stiffness of RVE II described above and filling volume fraction $f_\text {gran}^\text {congl}$, are surrounded by layers of newly grown bone tissue, with volume fraction $f_\text {bone}^\text {congl}$ and stiffness derived from the ultrasonic tests of Ashman and van Buskirk (1987). These coated spherical elements are assembled, in mutual contact, to a granular conglomerate with macropores, with volume fraction $\phi _\text {macro}^\text {congl}$, in-between. At the time of granule implantation, no bone tissue has been formed yet, and this initial configuration is characterized by $f_\text {bone}^\text {congl}\,=\,0$. For estimating the macroscopic stiffness tensor of the bone-scaffold conglomerate, $\mathbb {C}_\text {congl}$, the homogenization approach for an *n*-layered spherical inclusion proposed by Hervé and Zaoui (1993) is specialized for $n=1$ (relating to bone tissue), adapted for the case that the stiffness of this layer is transversally isotropic, see (Bertrand and Hellmich 2009), and further combined with a self-consistent homogenization scheme, in order to account for mutually interacting coated spheres with porous space in-between—in absence of any explicit “matrix phase”. This homogenization step is thus based on the composition and morphology of RVE III, as well as on the stiffness tensors of the granule material, $\mathbb {C}_\text {gran}$, accessible from stiffness homogenization across RVE II, of the bone tissue, $\mathbb {C}_\text {bone}$, and of the macropores, $\mathbb {C}_{\text {macro}\phi }$; the underlying mathematical framework is described at length in (Scheiner et al. 2016).

## Downscaling of stresses from macro- to microscale

The stiffness homogenization scheme for the herein investigated biomaterial scaffold for mandibular regeneration, described in Sect. 2.3, constitutes the basis for stress downscaling. For conciseness, the derivation of this (mathematically extensive) model is not repeated here in minute detail; instead, we focus on elaborating how stress downscaling is achieved, while assuming that the homogenized stiffness tensors on all observation scales, compare Fig. 1, namely the stiffness tensor of the nanoporous hydroxyapatite matrix, $\mathbb {C}_\text {polyHA}$, the stiffness tensor of the pre-cracked, mesoporous granule material, $\mathbb {C}_\text {gran}$, and the stiffness tensor of the bone-scaffold conglomerate, $\mathbb {C}_\text {congl}$, are known from the respective homogenization steps. In particular, we first clarify the mechanical input parameters the employed downscaling approach is based on (see Sect. 3.1), and present then briefly the three downscaling steps in terms of the underlying mathematical framework:

1. From the macroporous bone-scaffold conglomerate to the pre-cracked and mesoporous granules (see Sect. 3.2);
2. From the pre-cracked and mesoporous granules to the microporous, polycrystalline hydroxyapatite matrix (see Sect. 3.3); and
3. From the microporous, polycrystalline hydroxyapatite matrix to single, arbitrarily oriented hydroxyapatite crystal needles (see Sect. 3.4).

### Definition of mechanical input parameters

As for the underlying main elementary constituent, i.e. hydroxyapatite, the respective stiffness tensor, $\mathbb {C}_\text {HA}$, is defined via the bulk modulus, $k_\text {HA}$, and the shear modulus, $\mu _\text {HA}$, $\mathbb {C}_\text {HA}\,=\,3k_\text {HA}\mathbb {K}+2\mu _\text {HA}\mathbb {J}$, with $\mathbb {K}$ being the volumetric part of the fourth-order unit tensor $\mathbb {I}$, and $\mathbb {J}$ the corresponding deviatoric part, $\mathbb {K}+\mathbb {J}\,=\,\mathbb {I}$. Numerical values for $k_\text {HA}$ and $\mu _\text {HA}$ are found based on the experiments performed by Katz and co-workers (Katz and Ukraincik 1971; Gilmore and Katz 1982), who revealed the Young’s modulus and Poisson’s ratio of hydroxyapatite, $E_\text {HA}\,=\,114$ GPa and $\nu _\text {HA}\,=\,0.27$, see also (Hellmich and Ulm 2002; Hellmich et al. 2004). Through standard relations of continuum mechanics, $k\,=\,E/[3(1-2\nu )]$ and $\mu \,=\,E/[2(1+\nu )]$ (Mang and Hofstetter 2000), one finally obtains $k_\text {HA}\,=\,82.61$ GPa and $\mu _\text {HA}\,=\,44.88$ GPa. Furthermore, all pore spaces are assumed to be drained at all times, thus $\mathbb {C}_{\text {micro}\phi }\,=\,\mathbb {C}_{\text {meso}\phi }\,=\,\mathbb {C}_{\text {macro}\phi }\,=\,0$.

For defining the stiffness tensor of newly formed bone tissue, $\mathbb {C}_\text {bone}$, we consider the orthotropic stiffness tensor determined for mandibular bone by means of ultrasound measurements by Ashman and van Buskirk (1987), and follow then the strategy described in (Bertrand and Hellmich 2009) for converting the ultrasound-based, anisotropic stiffness tensor into the transversally isotropic stiffness tensor related to the bone tissue growing on granules, see also (Scheiner et al. 2016) for details, yielding

1 $$\begin{aligned}&\mathbb {C}^ {({\mathbf {e}}_r,{\mathbf {e}}_\vartheta ,{\mathbf {e}}_\varphi )}_ \text {bone}\nonumber \\&\quad {=}\left( \begin{array}{c@{\quad }c@{\quad }c@{\quad }c@{\quad }c@{\quad }c} 15.90 &{} 9.00 &{} 9.00 &{} 0 &{} 0 &{} 0\\ 9.00 &{} 21.74 &{} 10.70 &{} 0 &{} 0 &{} 0\\ 9.00 &{} 10.70 &{} 21.74 &{} 0 &{} 0 &{} 0\\ 0 &{} 0 &{} 0 &{} 11.04 &{} 0 &{} 0\\ 0 &{} 0 &{} 0 &{} 0 &{} 7.93 &{} 0\\ 0 &{} 0 &{} 0 &{} 0 &{} 0 &{} 7.93\\ \end{array} \right) \,\text {GPa}\,{,}\nonumber \\ \end{aligned}$$

with superscript $({\mathbf {e}}_r,{\mathbf {e}}_\vartheta ,{\mathbf {e}}_\varphi )$ indicating that this stiffness tensor is expressed in a spherical coordinate system, owing to the fact that in the particular case, bone tissue is added in form of a concentric shell on top of the spherical granules.

### From the macroporous bone-scaffold conglomerate to the pre- cracked, mesoporous granules (Fig. 1, hierarchical level III)

Based on the pioneering work of Hervé and Zaoui (1993), the aforementioned stiffness homogenization scheme for granular hydroxyapatite-based biomaterials, see Sect.  2.3, gives, on the one hand, access to the volumetric stress tensor of the granule material, $\varvec{\varepsilon }_\text {gran}^\text {congl,vol}$, in response to a macroscopically applied volumetric strain tensor ${\mathbf {E}}_\text {congl}^\text {vol}$,

2 $$\begin{aligned} \varvec{\sigma }_\text {gran}^\text {congl,vol}= \frac{3k_\text {gran} \overline{\Gamma _\text {gran,1}^k}}{{\mathcal {D}}_k} {\mathbf {E}}_\text {congl}^\text {vol}\,, \end{aligned}$$

and, on the other hand, to the deviatoric stress tensor of the granule material, $\varvec{\varepsilon }_\text {gran}^\text {congl,dev}$, in response to a macroscopic strain tensor representing pure shear, ${\mathbf {E}}_\text {congl}^\text {shear}$,

3 $$\begin{aligned} \varvec{\sigma }_\text {gran}^\text {congl,dev}= 2\mu _\text {gran} \left( \overline{\Gamma _\text {gran,1}^\mu } - \frac{21\overline{\Gamma _\text {gran,2}^\mu }}{5(1-2\nu _\text {gran})} \right) {\mathbf {E}}_\text {congl}^\text {shear}\,,\nonumber \\ \end{aligned}$$

where $k_\text {gran}$ is the bulk modulus, $\mu _\text {gran}$ the shear modulus, and $\nu _\text {gran}$ the Poisson’s ratio of the granule material. Furthermore, Eqs. (2) and (3) are governed by a number of material properties, namely $\overline{\Gamma _\text {gran,1}^k}$, $\overline{\Gamma _\text {gran,1}^\mu }$, $\overline{\Gamma _\text {gran,2}^\mu }$, and ${\mathcal {D}}_k$. These material properties are functions of the conglomerate’s composition (quantified by volume fractions $f_\text {gran}^\text {congl}$ and $f_\text {bone}^\text {congl}$), as well as the stiffness tensors of the conglomerate consisting of scaffold material and bone tissue, $\mathbb {C}_\text {congl}$, of the granule material, $\mathbb {C}_\text {gran}$, of the added bone matrix, $\mathbb {C}_\text {bone}$, and of the macropores, $\mathbb {C}_{\text {macro}\phi }$. The somewhat unusual form of the downscaling relations given by Eqs. (2) and (3) as well as of the lengthy mathematical expressions for $\overline{\Gamma _\text {gran,1}^k}$, $\overline{\Gamma _\text {gran,1}^\mu }$, $\overline{\Gamma _\text {gran,2}^\mu }$, and ${\mathcal {D}}_k$, given in detail in (Scheiner et al. 2016), result from the nature of the employed coated inclusion-problem of Hervé and Zaoui (1993), where, in contrast to the classical Eshelby-problem, the stresses and strains are *not* homogeneous throughout the inclusion.

Considering now that any strain (and, of course, also stress) tensor can be decomposed into volumetric and pure shear strain tensors, Eqs. (2) and (3) can be utilized for downscaling a general macroscopic strain tensor, ${\mathbf {E}}_\text {congl}$ to the corresponding stress tensor experienced by the granule material,

4 $$\begin{aligned} \begin{aligned} \varvec{\sigma }_\text {gran}^\text {congl}=&\,\, \frac{3k_\text {gran} \overline{\Gamma _\text {gran,1}^k}}{{\mathcal {D}}_k} \frac{\text {tr}\,{\mathbf {E}}_\text {congl}}{3}{\mathbf {I}}+ 2\mu _\text {gran}\\&\times \,\left( \overline{\Gamma _\text {gran,1}^\mu } -\frac{21\overline{\Gamma _\text {gran,2}^\mu }}{5(1-2\nu _\text {gran})} \right) \\&\times \,\left( {\mathbf {E}}_\text {congl}-\frac{\text {tr}\, {\mathbf {E}}_\text {congl}}{3}{\mathbf {I}}\right) \,, \end{aligned} \end{aligned}$$

where $\text {tr}$ is the trace operator, $\text {tr}\,{\mathbf {E}}_\text {congl}\,=\,E_{\text {congl},11}+E_{\text {congl},22}+E_{\text {congl},33}$, and ${\mathbf {I}}$ is the second-order unit tensor. Given that the underlying constitutive law on the observation scale of the bone-scaffold conglomerate is linear elastic, $\varvec{\Sigma }_\text {congl}\,=\,\mathbb {C}_\text {congl}:{\mathbf {E}}_\text {congl}$, Eq. (4) can be straightforwardly applied for also downscaling macroscopic stress tensors to the granule material level, through substituting ${\mathbf {E}}_\text {congl}$ by $(\mathbb {C}_\text {congl})^{-1}:\varvec{\Sigma }_\text {congl}$.

**Fig. 2:**
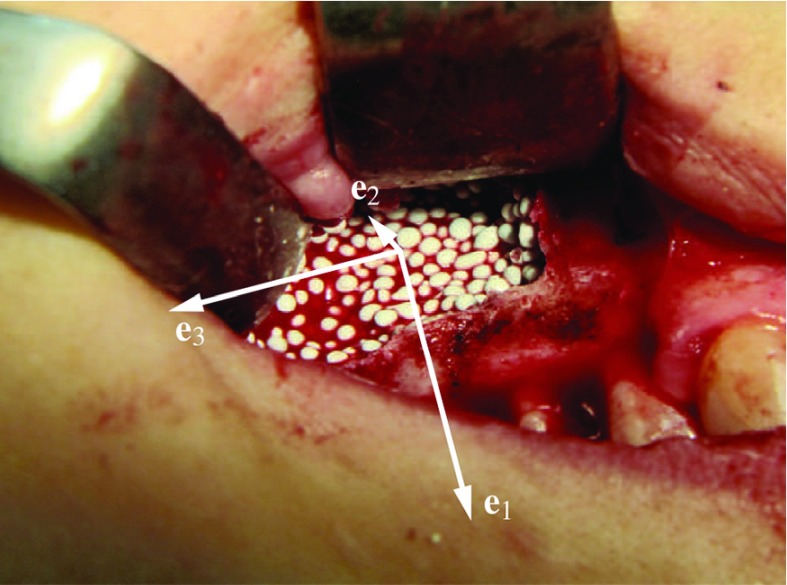
Image of the studied bone-scaffold conglomerate inserted into the upper jaw, based on which the macroscopic mechanical boundary conditions of the conglomerate consisting of bone tissue-coated granules are defined: load application occurs in direction **e** $_1$ (resembling mastication), deformation in direction **e** $_3$ is presumably prevented, the material is stress-free in direction **e** $_2$, the conglomerate material is furthermore free of shear stresses and strains

In the following, we want to exemplify stress downscaling, quantitatively, through prescribing macroscopically the predominant loading type expected for the studied biomaterial in physiological conditions. Typically, the biomaterial investigated in this paper is inserted from the buccal (exterior) mandible surface, and the granules-filled bone defect is covered afterwards by means of a bioresorbable membrane, see Fig. 2 (where the membrane has not yet been put in place). Considering that the macroscopic loading is usually prescribed in terms of stress component $\Sigma _{\text {congl,11}}\,<\,0$ (relating to mastication), and that the further mechanical boundary conditions for the bone-scaffold conglomerate are $E_{\text {congl},11}\,<\,0$, $E_{\text {congl,22}}\,>\,0$, $E_{\text {congl},33}\,=\,E_{\text {congl},12}\,=\,E_{\text {congl},13}\,=\,E_{\text {congl},33}\,=\,0$, $\Sigma _{\text {congl},33}\,<\,0$, and $\Sigma _{\text {congl,22}}\,=\,\Sigma _{\text {congl},12}\,=\,$ $\Sigma _{\text {congl},13}\,=\,0$ gives access, via the linear elastic constitutive law, to the corresponding macroscopic stress tensor:

5 $$\begin{aligned} \varvec{\Sigma _\text {congl}}= \left( \begin{array}{c} \Sigma _{\text {congl},11}\\ 0\\ \dfrac{3k_\text {congl}-2\mu _\text {congl}}{2(3k_\text {congl}+\mu _\text {congl})} \Sigma _{\text {congl},11}\\ 0\\ 0\\ 0\\ \end{array}\right) \,. \end{aligned}$$

**Fig. 3:**
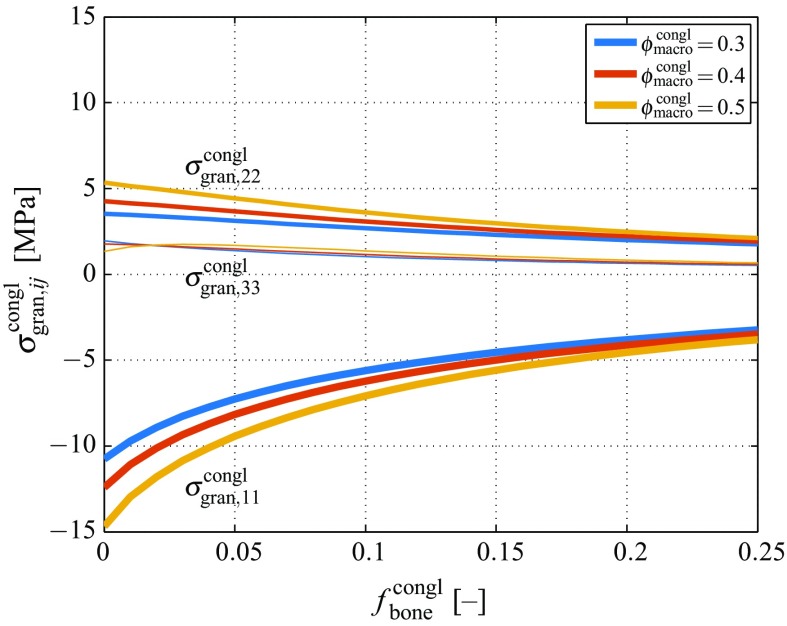
Non-zero components of the stress tensor of the granule material according to Eq. (4), for varying bone tissue volume fraction $f_\text {bone}^\text {congl}$, and under physiologically relevant macroscopic stress according to Eq. (5), with $\Sigma _{\text {congl},11}\,=\,-10\,$MPa

In the following, the macroscopic stress tensor component in direction of base vector ${\mathbf {e}}_1$ is set to $\Sigma _{\text {congl},11}\,=\,-10\,$MPa, while Eq. (5) gives access to the second non-zero component of the macroscopic stress tensor. Furthermore, the crack density parameter for quantifying the occurrence of cracks on hierarchical level II is set to $e=10$ (representing a low to moderate crack density), see Sect. 3.3 for further details. The downscaling relation given by Eq. (4) provides then the corresponding stress tensor experienced by the granule material, see Fig. 3, where also the effect of increasing bone volume fraction is illustrated. Notably, the macrocopically applied biaxial loading relates to a triaxial loading on one observation scale below.

### From the pre-cracked, mesoporous granules to the microporous hydroxyapatite polycrystal (Fig. 1, hierarchical level II)

For this downscaling step we can make use of classical strain and stress downscaling as defined in the framework of continuum micromechanics. As derived elsewhere, see e.g. (Zaoui 2002; Dormieux et al. 2006), continuum micromechanics allows to downscale strain tensors through the so-called concentration (or localization) tensors: for the present case, this implies that the strain tensor of the microporous hydroxyapatite polycrystal, $\varvec{\varepsilon }_\text {polyHA}^\text {gran}$, is related to the strain tensor of the granule material, $\varvec{\varepsilon }_\text {gran}^\text {congl}$, through

6 $$\begin{aligned} \varvec{\varepsilon }_\text {polyHA}^\text {gran}= \mathbb {A}_\text {polyHA}^\text {gran}: \varvec{\varepsilon }_\text {gran}^\text {congl}\,, \end{aligned}$$

where $\mathbb {A}_\text {polyHA}^\text {gran}$ is the strain concentration tensor of the microporous hydroxyapatite polycrystal. The latter is estimated by means of Eshelby’s matrix-inclusion problem (Eshelby 1957); considering the matrix-inclusion-type morphology discernible on hierarchical level II, compare Fig. 1, $\mathbb {A}_\text {polyHA}^\text {gran}$ is defined by (Deudé et al. 2002; Dormieux et al. 2004)

7 $$\begin{aligned} \begin{aligned}&\mathbb {A}_\text {polyHA}^\text {gran}=\,\,\bigg \{f_\text {polyHA}^\text {gran}\mathbb {I}+ \phi _\text {meso}^\text {gran}\nonumber \\&\qquad \times \left[ \mathbb {I}-\mathbb {P}_\text {sph}^\text {polyHA}: \left( \mathbb {C}_{\text {meso}\phi }- \mathbb {C}_\text {polyHA}\right) \right] ^{-1}+e\mathbb {Q}\bigg \} ^{-1}, \end{aligned}\\ \end{aligned}$$

where $\mathbb {P}_\text {sph}^\text {polyHA}$ is the fourth-order Hill tensor related to spherical inclusions embedded in a matrix exhibiting a stiffness tensor $\mathbb {C}_\text {polyHA}$, see (Eshelby 1957; Zaoui 2002) for how $\mathbb {P}_\text {sph}^\text {polyHA}$ is computed. Hence, in contrast to the situation in Sect.  3.2, we here employ the Eshelby’s classical matrix-inclusion problem (Eshelby 1957). Finally, *e* is the so-called crack density parameter (Budianksy and O’Connell 1976), $e\,=\,{\mathcal {N}}(r_\text {crack})^3$, with ${\mathcal {N}}$ as the number of cracks per volume, and $r_\text {crack}$ as the (average) crack radius, and $\mathbb {Q}$ is a tensor defined via the Poisson’s ratio of the microporous hydroxyapatite polycrystal, $\nu _\text {polyHA}$, through (Dormieux et al. 2004)

8 $$\begin{aligned} \mathbb {Q}= & {} \frac{16}{9}\frac{1-(\nu _\text {polyHA})^2}{1-2\nu _\text {polyHA}}\mathbb {K}\nonumber \\&+\frac{32}{45}\frac{(1-\nu _\text {polyHA}) (5-\nu _\text {polyHA})}{2-\nu _\text {polyHA}}\mathbb {J}\,, \end{aligned}$$

where $\mathbb {J}$ is the devatoric part of the fourth-order unit tensor $\mathbb {I}$, $\mathbb {J}\,=\,\mathbb {I}-\mathbb {K}$.

Linear elasticity on all hierarchical levels implies $\varvec{\varepsilon }_\text {gran}^\text {congl}\,=\,(\mathbb {C}_\text {gran})^{-1}:\varvec{\sigma }_\text {gran}^\text {congl}$, as well as $\varvec{\varepsilon }_\text {polyHA}^\text {gran}\,=\,(\mathbb {C}_\text {polyHA})^{-1}:\varvec{\sigma }_\text {polyHA}^\text {gran}$, so that the strain concentration relation of Eq. (6) can be transformed into a fully equivalent stress concentration relation of the format

9 $$\begin{aligned} \varvec{\sigma }_\text {polyHA}^\text {gran}= \mathbb {B}_\text {polyHA}^\text {gran}: \varvec{\sigma }_\text {gran}^\text {congl}\,, \end{aligned}$$

with the stress concentration tensor of the microporous hydroxyapatite matrix, $\mathbb {B}_\text {polyHA}^\text {gran}$, following from the strain concentration tensor $\mathbb {A}_\text {polyHA}^\text {gran}$, through

10 $$\begin{aligned} \mathbb {B}_\text {polyHA}^\text {gran}= \mathbb {C}_\text {polyHA}: \mathbb {A}_\text {polyHA}^\text {gran}: (\mathbb {C}_\text {gran})^{-1}\,. \end{aligned}$$

**Fig. 4:**
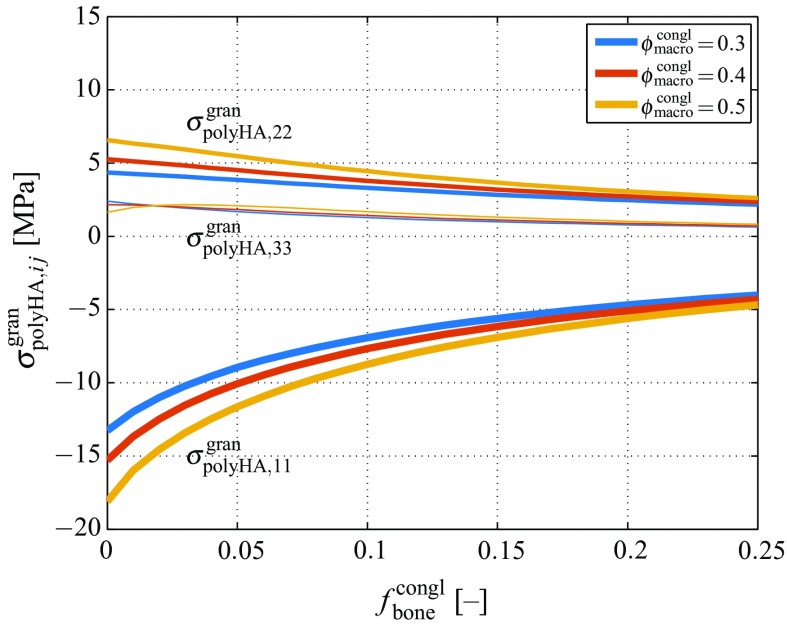
Non-zero components of the stress tensor of the microporous hydroxyapatite polycrystal according to Eqs. (4) and (9), for varying bone tissue volume fraction $f_\text {bone}^\text {congl}$, and under physiologically relevant macroscopic stress according to Eq. (5), with $\Sigma _{\text {congl},11}\,=\,-10\,$MPa

Downscaling of stresses according to Eq. (9) leads to further magnification of stresses; see Fig. 4 for the non-zero stress tensor components of the microporous granule material, obtained through application of Eq. (9) to the stresses depicted in Fig. 3.

### From the microporous hydroxyapatite polycrystal to the single hydroxyapatite crystal (Fig. 1, hierarchical level III)

**Fig. 5:**
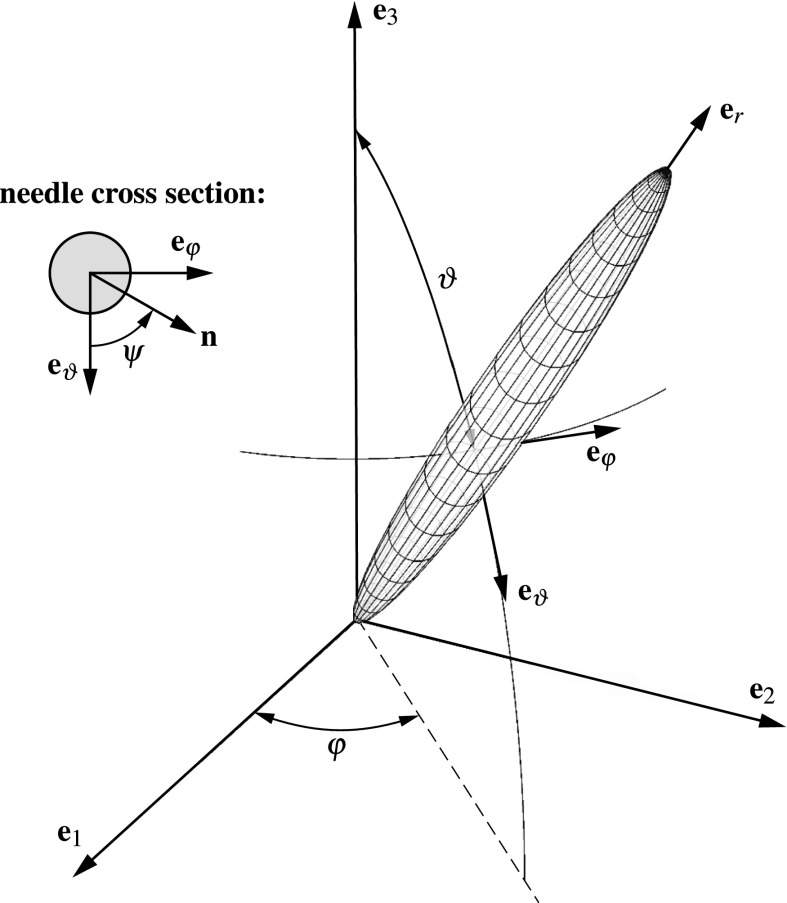
Cylindrical hydroxyapatite needle oriented along vector **e** $_r$, and inclined by the Euler angles $\vartheta$ and $\varphi$, with respect to the reference base frame defined through the unit vectors **e** $_1$, **e** $_2$, and **e** $_3$; the local base frame, defined by unit vectors **e** $_r$, **e** $_\vartheta$, and **e** $_\varphi$, is attached to the cylindrical inclusion; vector $\mathbf{n}$, oriented perpendicular to **e** $_r$, is further defined by angle $\psi$

The hydroxyapatite crystals making up the the microporous hydroxyapatite polycrystal are oriented arbitrarily, in all space directions, defined by in a spherical coordinate system by Euler angles $\vartheta$ and $\varphi$, see Fig. 5. Each orientation implies different stress levels occurring in the respective hydroxyapatite crystal, $\varvec{\sigma }_\text {HA}^\text {polyHA}\,=\,\varvec{\sigma }_\text {HA}^\text {polyHA}(\vartheta ,\varphi )$, for a given macroscopic loading. Making use of the orientation-dependent stress concentration tensor related to hydroxyapaytite needles, $\mathbb {B}_\text {HA}^\text {polyHA}(\vartheta ,\varphi )$, stress tensor $\varvec{\sigma }_\text {HA}^\text {polyHA}(\vartheta ,\varphi )$ follows as

11 $$\begin{aligned} \varvec{\sigma }_\text {HA}^\text {polyHA} (\vartheta ,\varphi )= \mathbb {B}_\text {HA}^\text {polyHA}(\vartheta ,\varphi ): \varvec{\sigma }_\text {polyHA}^\text {gran}\,, \end{aligned}$$

with $\varvec{\sigma }_\text {polyHA}^\text {gran}$ following from Eq. (9).

**Fig. 6:**
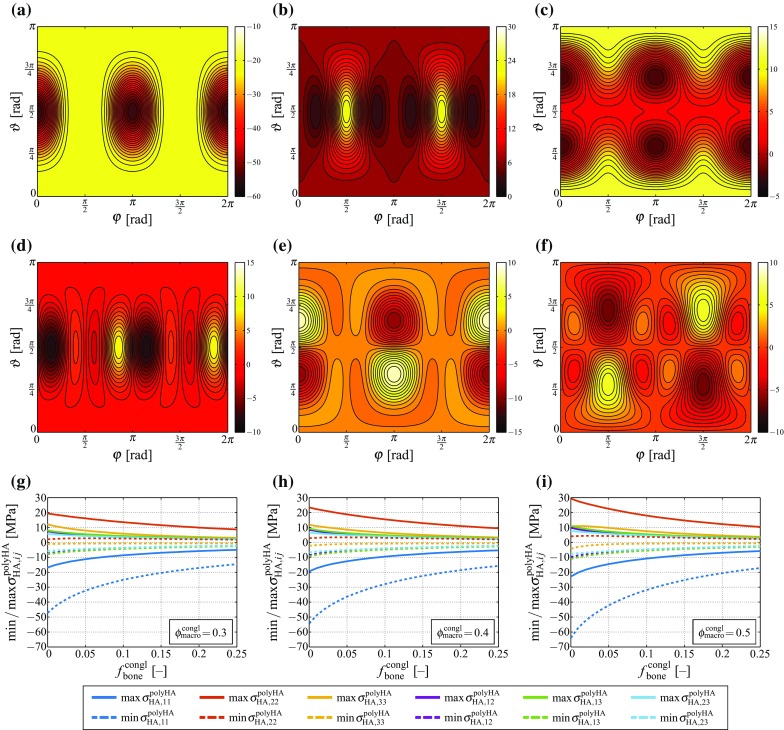
Components of the stress tensor of the hydroxapatite needles: components **a** $\sigma _{\text {HA},11}^\text {polyHA}$, **b** $\sigma _{\text {HA},22}^\text {polyHA}$, **c** $\sigma _{\text {HA},33}^\text {polyHA}$, **d** $\sigma _{\text {HA},12}^\text {polyHA}$, **e** $\sigma _{\text {HA},13}^\text {polyHA}$, and **f** $\sigma _{\text {HA},23}^\text {polyHA}$ as functions of the needle orientation (defined by angles $\vartheta$ and $\varphi$), for constant volume fractions of the macropores $\phi _\text {macro}^\text {congl}=0.4$ and of bone tissue $f_\text {bone}^\text {congl}=0$, when feeding the macroscopic stress tensor defined by Eq. (5) into the downscaling relations given by Eqs. (4), (9), and (11) with $\Sigma _{\text {congl},11}=-10\,$MPa; **g** – **i** minimum and maximum values of the stress tensor components for varying macroporosities and bone tissue volume fractions

Stress concentration tensor $\mathbb {B}_\text {HA}^\text {polyHA}(\vartheta ,\varphi )$ is defined analogously to Eq. (10),

12 $$\begin{aligned} \mathbb {B}_\text {HA}^\text {polyHA}(\vartheta ,\varphi )= \mathbb {C}_\text {HA}: \mathbb {A}_\text {HA}^\text {polyHA}(\vartheta ,\varphi ): \left( \mathbb {C}_\text {polyHA}\right) ^{-1}\,.\nonumber \\ \end{aligned}$$

The orientation-dependent strain concentration tensor $\mathbb {A}_\text {HA}^\text {polyHA}(\vartheta ,\varphi )$ is defined, according to (Fritsch et al. 2006), by

13 $$\begin{aligned}&\mathbb {A}_\text {HA}^\text {polyHA}(\vartheta ,\varphi ){=}\bigg [\mathbb {I}+ \mathbb {P}_\text {cyl}^\text {polyHA}(\vartheta ,\varphi ): \left( \mathbb {C}_\text {HA}{-}\mathbb {C}_\text {polyHA} \right) \bigg ]^{-1}\nonumber \\&\qquad \quad :\Bigg \{f_\text {HA}^\text {polyHA}\Bigg [ \int \limits _{\varphi =0}^{2\pi } \int \limits _{\vartheta =0}^\pi \bigg [\mathbb {I}+ \mathbb {P}_\text {cyl}^\text {polyHA}(\vartheta ,\varphi )\nonumber \\&\qquad \quad :\left( \mathbb {C}_\text {HA}- \mathbb {C}_\text {polyHA}\right) \bigg ]^{-1} \frac{\text {sin}\,\vartheta \,\text{ d }\vartheta \, \text{ d }\varphi }{4\pi }\Bigg ]\nonumber \\&\qquad \quad +\,\,\phi _\text {micro}^\text {polyHA} \left[ \mathbb {I}+ \mathbb {P}_\text {sph}^\text {polyHA}:\left( \mathbb {C}_{\text {micro}\phi }-\mathbb {C}_\text {polyHA} \right) \right] \Bigg \}^{-1},\nonumber \\ \end{aligned}$$

with $\mathbb {P}_\text {cyl}^\text {polyHA}(\vartheta ,\varphi )$ as the orientation-dependent Hill tensor relating to cylindrical inclusions embedded in the isotropic microporous hydroxyapatite polycrystal matrix with stiffness $\mathbb {C}_\text {polyHA}$, see (Eshelby 1957). The double integral in Eq. (13), expressing summation over all possible orientations, can be straightforwardly evaluated based on Stroud’s integration equations (Stroud 1971; Pichler et al. 2009). Notably, $\mathbb {A}_\text {HA}^\text {polyHA}(\vartheta ,\varphi )$ has been derived based on Eshelby’s famous matrix-inclusion problem (Eshelby 1957).

Feeding the composition-dependent stresses depicted in Fig. 4 into Eq. (11) reveals substantial (hydroxyapatite needle orientation-dependent) stress magnification effects, see Fig. 6. In particular, Fig. 6a–f show the variations between the different components of the hydroxyapatite stress tensor, for $\phi _\text {macro}^\text {congl}=0.4$ and $f_\text {bone}^\text {congl}=0$. In order to also highlight the extreme values of the stress tensor components as functions of the scaffold composition, Fig. 6g–i show the minimum and maximum values of all stress tensor components over all hydroxyapatite needle orientations, see also Table 1 for an overview of the observed maximum and minimum stress tensor components. Notably, minimum stresses stresses (i.e. compressive stresses with maximum magnitude) always occur in direction ${\mathbf {e}}_1$, while maximum stresses (i.e. tensile stresses with maximum magnitude) always occur in direction ${\mathbf {e}}_2$.

**Table 1:** Minimum and maximum values of stress tensor components $\sigma _{\text {HA},ij}^\text {polyHA}$, occurring when prescribing the macroscopic stress tensor given by Eq. (5), with $\Sigma _{\text {congl},11}\,=\,-10$ MPa, compare Fig. 6g – i

$\phi _\text {macro}^\text {congl}$	$\min {\sigma _{\text {HA},ij}^\text {polyHA}}$ (MPa)	$\max {\sigma _{\text {HA},ij}^\text {polyHA}}$ (MPa)
0.3	$-47.24$	19.41
0.4	$-54.31$	23.45
0.5	$-63.99$	29.38

## Estimates for the macroscopic strength of hydroxyapatite-based granular biomaterials

### Failure criterion suitable for hydroxyapatite needles

Based on the three-step scheme presented in Sect. 3, a macroscopically applied mechanical loading, prescribed in terms of macroscopic strains ${\mathbf {E}}_\text {congl}$ or macroscopic stresses $\varvec{\Sigma }_\text {congl}$, were downscaled to the corresponding stress state experienced by a single, arbitrarily oriented hydroxyapatite needle, $\varvec{\sigma }_\text {HA}^\text {polyHA}(\vartheta ,\varphi )$.

From (Fritsch et al. 2009a, b), we adopt that hydroxyapatite needle failure is governed by the normal stress in needle direction,

14 $$\begin{aligned} \sigma _{\text {HA},rr}(\vartheta ,\varphi )= {\mathbf {e}}_r(\vartheta ,\varphi )\cdot \varvec{\sigma }_\text {HA}^\text {polyHA} (\vartheta ,\varphi )\cdot {\mathbf {e}}_r(\vartheta ,\varphi )\,,\nonumber \\ \end{aligned}$$

and by the shear stress in planes orthogonal to the needle direction,

**Table 2:** Iteration scheme for deriving the macroscopic loading of the biomaterial inducing quasi-brittle failure in the most unfavorably stressed hydroxyapatite needle

Iteration steps	
1.	Choice of initial value for $\Sigma _{\text {congl},11}$.
2.	Computation of macroscopic stress tensor according to Eq. (5).
3.	Downscaling of macroscopic stress tensor to the level of hydroxyapatite needles as function of the needle orientation, $\varvec{\sigma }_\text {HA}^\text {polyHA}(\vartheta ,\varphi )$, $\vartheta \,=\,0\ldots \pi$, $\varphi \,=\,0\ldots 2\pi$, by means of Eqs. (2–13).
4.	Calculation of the corresponding normal and shear stress component experienced by single hydroxyapatite needles, for any needle orientation, $\vartheta \,=\,0\ldots \pi$, $\varphi \,=\,0\ldots 2\pi$, and for any tangential plane, $\psi \,=\,0\ldots 2\pi$, by means of Eqs. (14–18).
5.	Evaluation of the failure criterion given by Eqs. (19) and (20), respectively:
	–If ${\mathfrak {f}}_\text {HA}(\varvec{\Sigma }_\text {congl})\,<\,0$, then $|\Sigma _{\text {congl},11}|$ is increased; return to step 2.
	–If ${\mathfrak {f}}_\text {HA}(\varvec{\Sigma }_\text {congl})\,=\,0$, then the load iteration is completed, and the current magnitude for $\Sigma _{\text {congl},11}$ induces failure of the material.
	–If ${\mathfrak {f}}_\text {HA}(\varvec{\Sigma }_\text {congl})\,>\,0$, then $|\Sigma _{\text {congl},11}|$ is decreased; return to step 2.

15 $$\begin{aligned} \sigma _{\text {HA},rn}(\vartheta ,\varphi ,\psi )= {\mathbf {e}}_r(\vartheta ,\varphi )\cdot \varvec{\sigma }_\text {HA}^\text {polyHA}(\vartheta ,\varphi )\cdot {\mathbf {n}}(\vartheta ,\varphi ,\psi )\,.\nonumber \\ \end{aligned}$$

In Eqs. (14) and (15), ${\mathbf {e}}_r(\vartheta ,\varphi )$ denotes the vector defining the direction of a particular needle, i.e. the base vector of the employed spherical coordinate system in radial direction, while vector ${\mathbf {n}}(\vartheta ,\varphi ,\psi )$ denotes the direction orthogonal to ${\mathbf {e}}_r(\vartheta ,\varphi )$, additionally governed by angle $\psi$, compare Fig. 5. As for definition of vector ${\mathbf {n}}(\vartheta ,\varphi ,\psi )$, we consider that the needle orientation-dependent base vectors $({\mathbf {e}}_r,{\mathbf {e}}_\vartheta ,{\mathbf {e}}_\varphi )$ are defined in the Cartesian base system $({\mathbf {e}}_1,{\mathbf {e}}_2,{\mathbf {e}}_3)$ as

16 $$\begin{aligned} \begin{aligned} {\mathbf {e}}_r=&\left( \begin{array}{ccc} \sin {\vartheta }\cos {\varphi },&\sin {\vartheta }\sin {\varphi },&\cos {\vartheta } \end{array}\right) ^\text {T},\\ {\mathbf {e}}_\vartheta =&\left( \begin{array}{ccc} \cos {\vartheta }\cos {\varphi },&\cos {\vartheta }\sin {\varphi },&-\sin {\vartheta } \end{array}\right) ^\text {T},\\ {\mathbf {e}}_\varphi =&\left( \begin{array}{ccc} -\sin {\varphi },&\cos {\varphi },&0 \end{array}\right) ^\text {T}, \end{aligned} \end{aligned}$$

and that the transformation tensor ${\mathbf {Q}}$ from base system $({\mathbf {e}}_1,{\mathbf {e}}_2,{\mathbf {e}}_3)$ to base system $({\mathbf {e}}_r,{\mathbf {e}}_\vartheta ,{\mathbf {e}}_\varphi )$ reads as

17 $$\begin{aligned} \begin{aligned} {\mathbf {Q}}=&\, \left( {\mathbf {e}}_r;{\mathbf {e}}_\varphi ; {\mathbf {e}}_\vartheta \right) ^\text {T}\\ =&\,\left( \begin{array}{ccc} \sin {\vartheta }\cos {\varphi } &{} \sin {\vartheta }\sin {\varphi } &{} \cos {\vartheta }\\ \cos {\vartheta }\cos {\varphi } &{} \cos {\vartheta }\sin {\varphi } &{} -\sin {\vartheta }\\ -\sin {\varphi } &{} \cos {\varphi } &{} 0 \end{array}\right) \,. \end{aligned} \end{aligned}$$

Then, vector ${\mathbf {n}}(\vartheta ,\varphi ,\psi )$, dependent on angles $\vartheta$, $\varphi$, and $\psi$, and expressed in a Cartesian base frame, follows as

18 $$\begin{aligned} {\mathbf {n}}(\vartheta ,\varphi ,\psi )= {\mathbf {Q}}^\text {T}\cdot \left( \begin{array}{c} 0 \\ \cos {\psi } \\ \sin {\psi } \end{array}\right) \,. \end{aligned}$$

As furthermore proposed in (Fritsch et al. 2009a, b), the failure criterion for a single hydroxyapatite needles takes into account both tensile strength $\sigma _\text {HA}^\text {ult,t}$ and shear strength $\sigma _\text {HA}^\text {ult,s}$. The two strength values are accessible through analyzing the experiments of Akao et al. (1981) and Shareef et al. (1993), revealing $\sigma _\text {HA}^\text {ult,t}\,=\,52.2$ MPa and $\sigma _\text {HA}^\text {ult,s}\,=\,80.3$ MPa, see (Fritsch et al. 2009a). Mathematically, the failure surface related to the hydroxyapatite needles reads as

19 $$\begin{aligned} {\mathfrak {f}}_\text {HA}(\varvec{\sigma }_\text {HA}^ \text {polyHA})= & {} \max \limits _{\vartheta ,\varphi } \Bigg (\frac{\sigma _\text {HA}^\text {ult,t}}{\sigma _\text {HA}^\text {ult,s}} \max \limits _\psi \left| \sigma _{\text {HA},rn}^\text {polyHA} (\vartheta ,\varphi ,\psi )\right| \nonumber \\&+\sigma _{\text {HA},rr}^\text {polyHA} (\vartheta ,\varphi )\Bigg )- \sigma _\text {HA}^\text {ult,t}=0\,. \end{aligned}$$

Eq. (19) takes into account that for each needle orientation the angle $\psi$ inducing the maximum tangential stress must be found, as well as the needle orientation inducing the maximum value for the combination of normal and tangential stresses. For this purpose, angles $\vartheta$, $\varphi$, and $\psi$ are varied between $\vartheta \,=\,0\ldots \pi$, $\varphi \,=\,0\ldots 2\pi$, and $\psi \,=\,0\ldots 2\pi$. Substituting into Eq. (19) the relation between $\varvec{\sigma }_\text {HA}^\text {polyHA}$ and $\varvec{\Sigma }_\text {congl}$, according to the downscaling scheme elaborated in Sect. 3 allows to alternatively express the failure criterion in terms of macroscopic stresses,

20 $$\begin{aligned} {\mathfrak {f}}_\text {HA}(\varvec{\Sigma }_\text {congl})=0\,. \end{aligned}$$

### Computation of composition-dependent macroscopic loading inducing hydroxyapatite needle-failure

**Fig. 7:**
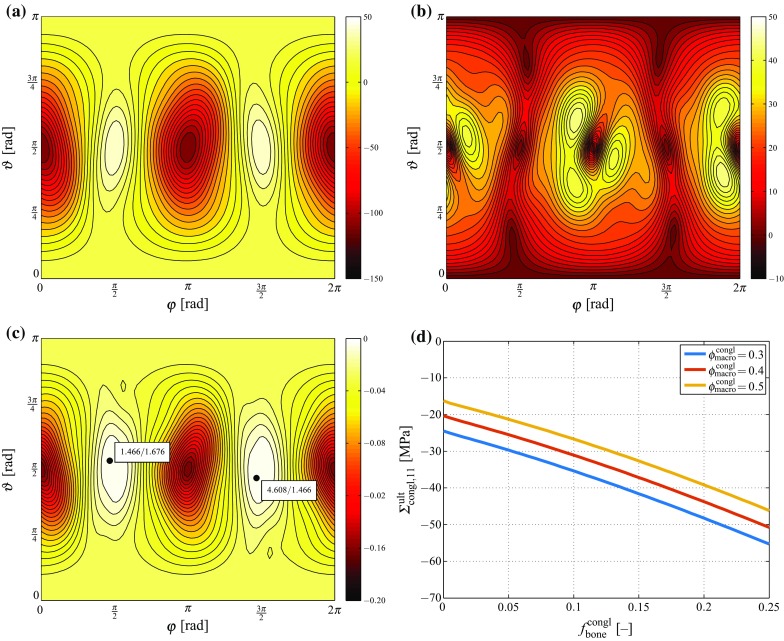
**a** Normal stress component $\sigma _{\text {HA},rr}^\text {polyHA}(\vartheta ,\varphi )$, **b** maximum shear stress component $\max \limits _\psi |\sigma _{\text {HA},rn}^\text {polyHA}(\vartheta ,\varphi ,\psi )|$, and **c** corresponding values of the failure function ${\mathfrak {f}}_\text {HA}(\varvec{\Sigma }_\text {congl})$, evaluated for $\phi _\text {macro}^\text {congl}=0.4$, $f_\text {bone}^\text {congl}=0$, and $\Sigma _{\text {congl},11}=-15.15$ MPa, as functions of the needle orientation; in **c**, the needle orientations at which ${\mathfrak {f}}_\text {HA}(\varvec{\Sigma }_\text {congl})=0$, are indicated; **d** the macroscopic, material failure-inducing stress tensor component $\Sigma _{\text {congl},11}$, as function of $\phi _\text {macro}^\text {congl}$ and of $f_\text {bone}^\text {congl}$

The failure criterion presented in Sect. 4.1, for estimating the macroscopic loading that leads to quasi-brittle failure of the most unfavorably stressed hydroxyapatite needle, is evaluated in an iterative manner, see Table 2. Let us e.g. consider, for the sake of demonstration, a biomaterial configuration defined by $\phi _\text {macro}^\text {congl}\,=\,0.4$ and $f_\text {bone}^\text {congl}\,=\,0$. Then, the iterative approach sketched in Table 2 reveals that a macroscopic stress tensor with non-zero components $\Sigma _{\text {congl},11}\,=\,-20.31$ MPa and $\Sigma _{\text {congl},33}\,=\,-3.09$ MPa is related to ${\mathfrak {f}}_\text {HA}(\varvec{\Sigma }_\text {congl})\,=\,0$, thus inducing failure of the most unfavorably stressed hydroxyapatite needle. The stress state of the latter is illustrated in Fig. 7a and b, in terms of the normal and maximum shear stress components, as functions of the needle orientation. The corresponding values of the failure function ${\mathfrak {f}}_\text {HA}(\varvec{\Sigma }_\text {congl})$, as obtained through insertion of the orientation-dependent normal and maximum shear stress components into the failure criterion, is again a function of the needle orientation, as depicted in Fig. 7(c). In this figure, the needle orientations that are actually evoking the most unfavorable stress states for the aforementioned macroscopic loading inducing ${\mathfrak {f}}_\text {HA}(\varvec{\Sigma }_\text {congl})\,=\,0$, are indicated, namely $(\varphi \,=\,1.466,\vartheta \,=\,1.676)$ and $(\varphi \,=\,4.608,\vartheta \,=\,1.466)$, respectively; or $(\varphi \,=\,84.00^\circ ,\,\vartheta \,=\,96.03^\circ )$ and $(\varphi \,=\,264.03^\circ$, $\vartheta \,=\,84.00^\circ )$, respectively.

Furthermore, the magnitude of the macroscopic, material failure-inducing loading increases with increasing bone volume fraction ($f_\text {bone}^\text {congl}$), as well as with decreasing volume fraction of the macropores ($\phi _\text {macro}^\text {congl}$), see Fig. 7d.

## Development of failure-inducing macroscopic stresses during bone regeneration

Once immersed into its targeted physiological environment, i.e. the immediate vicinity of (mandibular) bone tissue, the studied biomaterial undergoes compositional changes, due to two distinct mechanisms. On the one hand, the granules become coated with a layer of newly forming bone tissue whose thickness is growing with time. Presuming that the growth of bone tissue occurs linearly over time (Cancedda et al. 2007), the following bone volume fraction evolution law can be deduced:

21 $$\begin{aligned} f_\text {bone}^\text {congl}= \left[ \frac{\left( r_\text {gran}+k_\text {growth}t\right) ^3}{r_\text {gran}^3}-1\right] f_\text {gran}^\text {congl}\,, \end{aligned}$$

where *t* is the time after placing the scaffold in a bony environment, $k_\text {growth}$ is the bone growth rate, and $r_\text {gran}$ is the radius of the granules. For the presently studied material, the typical bone growth rate is $k_\text {growth}=4\pm 3\,\upmu$m/week (Scheiner et al. 2016). The time-dependent bone tissue volume fraction defined by Eq. (21) enters, as given in great detail in (Scheiner et al. 2016), the material functions $\overline{\Gamma _\text {gran,1}^k}$, $\overline{\Gamma _\text {gran,1}^\mu }$, $\overline{\Gamma _\text {gran,2}^\mu }$, and ${\mathcal {D}}_k$ appearing in Eqs. (2–4). In this way, Eq. (21) induces time-dependency in the strength of the investigated conglomerate material. In the same way, Eq. (21) induces time-dependencies in the homogenized stiffness expressions relating to the RVEs depicted in Fig. 1, as given in (Scheiner et al. 2016).

On the other hand, the contact of the bone-coated granules with physiological solution also leads to resorption of hydroxyapatite. As is known from analysis of X-ray microtomography scans, this resorption process occurs via growth of the micropores (Czenek et al. 2014),

22 $$\begin{aligned} \phi _\text {micro}^\text {polyHA}= \phi _{\text {micro},0}^\text {polyHA}+k_\text {res}t\,, \end{aligned}$$

with $\phi _{\text {micro},0}^\text {polyHA}$ as the microporosity before resorption sets in, and $k_\text {res}$ as scaffold resorption rate. Notably, $\phi _\text {micro}^\text {polyHA}$ enters stress and strain downscaling at Eq. (13) of this paper, thus influencing strain concentration tensor$\mathbb {A}_\text {HA}^\text {polyHA}(\vartheta ,\varphi )$, and therefore again the time-dependent strength and stiffness properties of the considered hierarchical biomaterial. In vitro studies have shown that the resorption rate may be as high as $k_\text {res}\,=\,0.016\,$week$^{-1}$, depending on the actual composition of the hosting medium (Scheiner et al. 2016).

**Table 3:** Values chosen for model input parameters, in order to study corresponding variations in the macroscopic failure load of bone tissue-coated hydroxyapatite-based scaffolds

Model input parameter	Unit	Numerical value (s)
Initial microporosity $\phi _{\text {micro},0}^\text {polyHA}$	(–)	0.445
Mesoporosity $\phi _\text {meso}^\text {gran}$	(–)	0.189
Macroporosity $\phi _\text {macro}^\text {congl}$	(–)	0.3, 0.4, 0.5
Crack density parameter *e*	(–)	0, 10, 25
Granule radius $r_\text {gran}$	($\upmu$m)	300, 500, 1000
Bone formation rate $k_\text {form}$	($\upmu$m/week)	4, 7, 10
Scaffold resorption rate $k_\text {res}$	(week$^{-1}$)	0, 0.008, 0.016

**Fig. 8:**
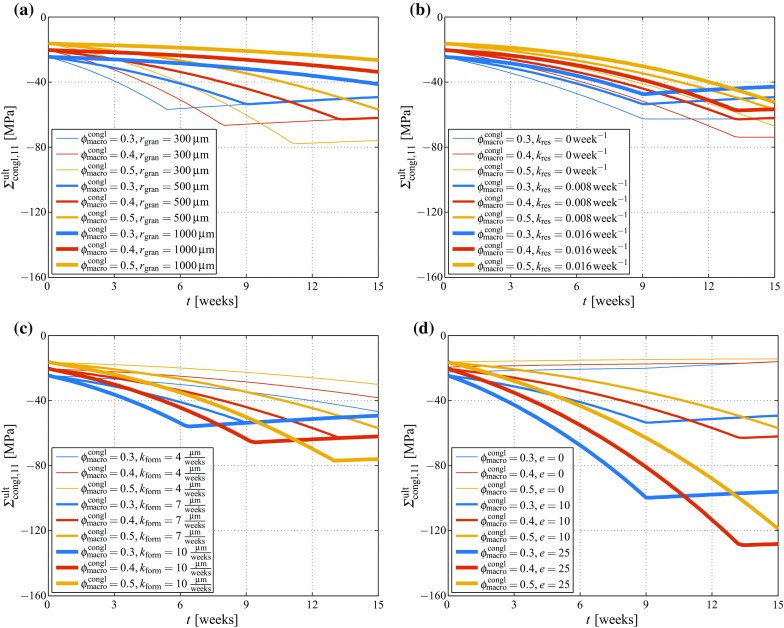
Development of $\Sigma _{\text {congl},11}^\text {ult}$ over time due to bone ingrowth and scaffold resorption, as function of the macroporosity $\phi _\text {macro}^\text {congl}$, $\phi _\text {macro}^\text {congl}=\{0.3,0.4,0.5\}$, as well as of **a** the granule radius $r_\text {gran}$, $r_\text {gran}=\{300,\,500,\,1000\,\upmu \text {m}\}$, **b** the scaffold resorption rate $k_\text {res}$, $k_\text {res}=\{0,0.008,\,0.016\,\text {week}^{-1}\}$, **c** the bone formation rate $k_\text {form}$, $k_\text {form}=\{4,\,7,\,10\,\upmu \text {m/week}\}$, and **d** the crack density parameter *e*, $e=\{0,10,25\}$

In the following, a series of parametric studies are presented, for elucidating the effects of model parameter variations on the predicted development of the macroscopic failure-inducing stress tensor component in direction ${\mathbf {e}}_1$, $\Sigma _{\text {congl},11}^\text {ult}$. For the sake of clarity, all model input parameters (and the variations considered for the numerical studies presented in this paper) are summarized in Table 3. The accordingly performed simulations highlight strong sensitivites of the macroscopic loading that causes failure of the hydroxyapatite needles, on the considered parameter variations, see Fig. 8. As regards the granule radius, it is striking that decreasing the radius initially implies accelerating the bone ingrowth-related increase of the load-carrying capacity, see Fig. 8a. However, the smaller the granule radius the sooner the complete pore space is filled with bone tissue, which is indicated by a peak of $\Sigma _{\text {congl},11}^\text {ult}$ (the actual magnitude of which depends on the macroporosity), followed by prolonged decrease of the load-carrying capacity, caused by scaffold resorption. Furthermore, it is interesting to note that the granule radius influences strongly the development of $\Sigma _{\text {congl},11}^\text {ult}$ directly after placing the granules in the targeted physiological environment (initiating scaffold resorption and bone formation), while the actual value of $\Sigma _{\text {congl},11}^\text {ult}$ at a later point of time is only governed by the macroporosity. A high macroporosity supports the strength development at mature bone regeneration states since it provides substantial space for newly added, load-carrying bone tissue; but it compromises strength before onset of bone regeneration as well as at early regeneration states as then a comparably low amount of bone-scaffold conglomerate needs to carry all (or most) of the loading.

The scaffold resorption rate, in turn, governs both the load-carrying capacity of the bone-coated granules when the macroporosity is completely filled with new bone tissue, as well as the long-term development afterwards. Zero resorption implies that the value of $\Sigma _{\text {congl},11}^\text {ult}$ is maintained at a constant (maximum) level (related to complete filling of the pore space by bone matrix), whereas a non-zero resorption rate causes a long-term decrease of $\Sigma _{\text {congl},11}^\text {ult}$ after reaching the aforementioned maximum value, see Fig. 8b. It should be noted that this long-term decrease is caused by assuming that bone growth merely occurs on the outer surface of the (bone-covered) granules. However, in reality, it can be assumed that after substantial resorption of the hydroxyapatite crystals the physiological solution enters the micro- and mesopore spaces, leading eventually also to bone formation within the granule body. The omission of this potential additional bone formation effect can be deemed as limitation of our model.

Similar to the granule radius, the bone formation rate only influences the time span until the whole macropore space is filled with bone tissue; the long-term development of the load-carrying capacity is unaffected, see Fig. 8c.

Remarkable effects are revealed when varying the crack density parameter *e*, see Fig. 8d. In particular, decreasing the crack density parameter leads to a significantly increased stiffness of the granule material. Considering that in a composite material (such as the material studied in this paper) stiffer constituents attract larger fractions of a macroscopically applied loading than softer constituents, which attract lower fractions of the macroscopic loading, a granule material containing less cracks hence transfers higher stresses to the hydroxyapatite needles than those with more cracks. This eventually implies that increasing the crack density in the granule material leads to an increased load-carrying capacity. This possibly counterintuitive conclusion straightforwardly suggests that future extensions of the here presented model should comprise formulation of a failure criterion related to the bone tissue as well, in order to improve the significance of the model-predicted load-carrying capacity.

## Discussion and concluding remarks

In this paper, a continuum micromechanics-based model was presented for estimating the macroscopic loading acting onto a hydroxyapatite-based granular biomaterial, developed for application as bone replacement material (in mandibular bone), that leads to quasi-brittle failure of the material’s main constituent, i.e. hydroxyapatite crystals. The parametric studies presented in Sect. 5 show how the load-carrying capacity of the studied biomaterial develops over time once placed in the targeted physiological environment, i.e. the immediate vicinity of mandibular bone, considering the growth of new bone tissue on the surface of the scaffold material, and resorption of the hydroxyapatite needles. Thereby, main emphasis was on highlighting the influence of specific design parameters of the production process, e.g. the exact chemical composition of the biomaterial might influence the rates of bone ingrowth and scaffold resorption, the crack density may be related to the production process, and the macroporosity can be tuned based on the packing density of the granules. From a practical point of view, the presented modeling approach allows to determine from when onwards a particular area of the mandible including an implant composed of the studied biomaterial can be used for mastication if the mechanical loading acting onto this mandibular region is approximately known, e.g. from Finite Element simulations (Korioth et al. 1992; Meijer et al. 1993; Choi et al. 2005; Hellmich et al. 2008; Bevilacqua et al. 2011).

However, the simulation results also point out two model restrictions. On the one hand, the kind of counterintuitive observation was made that a severely cracked granule material implies that the bone tissue growing on the granule surface attracts most of themacroscopically applied stress. Thus, our model suggests that increasing the crack density leads to an increasing load-carrying capacity, owing to the fact that the employed failure criterion considers only the most unfavorably stressed hydroxyapatite needle contained in the granules, neglecting however the stress experienced by the newly formed bone tissue. On the other hand, our model does not consider that the dissolution of hydroxyapatite needles would eventually lead to morphological changes in the microporous hydroxyapatite matrix (hierarchical level I in compare Fig. 1), implying that physiological solution could enter the micropore space, facilitating there bone tissue formation. Given that the described model inadequacies become relevant only after a certain (not yet quantifiable) time span, but not directly after scaffold implantation, leads to the conclusion that our model is particularly accurate for early-age bone-scaffold conglomerates (with respect to the time instant when the granules are placed into the targeted physiological environment), while the prediction accuracy presumably diminishes over time. This restriction constitutes the basis for reasonable future research directions.

Finally, it is also important to discuss the relevance of traditional fracture mechanics approaches, typically focusing on the prediction of crack propagation, thus assuming the existence of an initial crack (Müller et al. 2002; Näser et al. 2007; Kolednik et al. 2010; Ott et al. 2010), in the context of the material studied in this paper. Actually, it seems to be a worthwhile subject of future research actitivities to extend the micromechanics-based assessment of specific, microscopically sized material constituents (as demonstrated in this paper) towards traditional fracture mechanics, see e.g. (Pichler et al. 2007; Pichler and Dormieux 2009b, a). Such extension would be particularly relevant for mature bone-scaffold conglomerates containing already a substantial amount of bone tissue, allowing for studying the effects of crack emergence and propagation in the bone tissue—given that a respective failure criterion has been formulated, see e.g. (Fritsch et al. 2009b)—as well as of biologically driven crack healing.

## Abbreviations

gran: Granule material (RVE II)
$\mu$CT: Micro-computed tomography
polyHA: Hydroxyapatite polycrystal (RVE I)
RVE: Representative volume element
congl: Conglomerate of granules coated with bone tissue (RVE III)

## Latin symbols

$\mathbb {A}_\text {HA}^\text {polyHA}$: Fourth-order strain concentration tensor of hydroxyapatite crystal needles in hydroxyapatite polycrystal
$\mathbb {A}_\text {polyHA}^\text {gran}$: Fourth-order strain concentration tensor of hydroxyapatite polycrystal in granule material
$\mathbb {B}_\text {HA}^\text {polyHA}$: Fourth-order stress concentration tensor of hydroxyapatite crystal needles in hydroxyapatite polycrystal
$\mathbb {B}_\text {polyHA}^\text {gran}$: Fourth-order stress concentration tensor of hydroxyapatite polycrystal in granule material
$\mathbb {C}_\text {bone}$: Fourth-order stiffness tensor of bone tissue
$\mathbb {C}_\text {gran}$: Fourth-order stiffness tensor of granular material
$\mathbb {C}_{\text {H}_2\text {O}}$: Fourth-order stiffness tensor of water
$\mathbb {C}_\text {HA}$: Fourth-order stiffness tensor of hydroxyapatite crystals
$\mathbb {C}_{\text {macro}\phi }$: Fourth-order stiffness tensor of macropores
$\mathbb {C}_{\text {meso}\phi }$: Fourth-order stiffness tensor of mesopores
$\mathbb {C}_{\text {micro}\phi }$: Fourth-order stiffness tensor of micropores
$\mathbb {C}_\text {polyHA}$: Fourth-order stiffness tensor of hydroxyapatite polycrystal
$\mathbb {C}_\text {congl}$: Fourth-order stiffness tensor of bone-scaffold conglomerate
$${\mathcal {D}}_k$$: Material parameter
*d*: Characteristic length of an inhomogeneity within a representative volume element
*e*: Crack density parameter
${\mathbf {e}}_1,{\mathbf {e}}_2,{\mathbf {e}}_3$: Unit base vectors of a Cartesian base system
${\mathbf {e}}_r,{\mathbf {e}}_\vartheta ,{\mathbf {e}}_\varphi$: Unit base vectors of a spherical coordinate system
$E_\text {HA}$: Young’s modulus of hydroxyapatite crystals
${\mathbf {E}}_\text {congl}$: Second-order strain tensor of the bone-scaffold conglomerate
$$E_{\text {congl},ij}$$: Components of $${\mathbf {E}}_\text {congl}$$ ($$i,j=1,2,3$$)
${f}_\text {bone}^\text {congl}$: Volume fraction of bone tissue within bone-scaffold conglomerate
${f}_\text {gran}^\text {congl}$: Volume fraction of the granules within bone-scaffold conglomerate
$f_\text {HA}^\text {polyHA}$: Volume fraction of the hydroxyapatite needles within hydroxyapatite polycrystal
$f_\text {polyHA}^\text {gran}$: Volume fraction of the microporous hydroxyapatite matrix within granule material
${\mathfrak {f}}(\varvec{\sigma })$: Failure function formulated in terms of stress tensor $\varvec{\sigma }$
${\mathbf {I}}$: Second-order unit tensor
$\mathbb {I}$: Fourth-order unit tensor
$\mathbb {J}$: Deviatoric part of the fourth-order unit tensor
$k_\text {gran}$: Bulk modulus of the granule material
$k_\text {growth}$: Formation rate of bone tissue
$k_{\text {H}_2\text {O}}$: Bulk modulus of water
$k_\text {HA}$: Bulk modulus of the hydroxyapatite crystals
$k_\text {res}$: Resorption rate of hydroxyapatite crystals
$k_\text {congl}$: Bulk modulus of the bone-scaffold conglomerate
$\mathbb {K}$: Volumetric part of the fourth-order unit tensor
$\ell$: Characteristic length of a representative volume element
${\mathcal {L}}$: Characteristic length of a structure made up the material defined on the level of a representative volume element
${\mathbf {n}}$: Vector oriented perpendicular to unit base vector ${\mathbf {e}}_r$
$\mathbb {P}_\text {cyl}^\text {polyHA}$: Fourth-order Hill tensor of cylindrical inclusions in a matrix with stiffness $\mathbb {C}_\text {polyHA}$
$\mathbb {P}_\text {sph}^\text {polyHA}$: Fourth-order Hill tensor of spherical inclusions in a matrix with stiffness $\mathbb {C}_\text {polyHA}$
${\mathbf {Q}}$: Second-order transformation tensor
$\mathbb {Q}$: Fourth-order tensor defined through Poisson’s ratio $\nu _\text {polyHA}$
$$r_\text {gran}$$: Granule radius
*t*: Time variable

## Greek symbols

$$\overline{\Gamma _\text {gran,1}^k}$$: Material parameter
$$\overline{\Gamma _\text {gran,1}^\mu }$$: Material parameter
$$\overline{\Gamma _\text {gran,2}^\mu }$$: Material parameter
$\varvec{\varepsilon }_\text {HA}^\text {polyHA}$: Second-order strain tensor of hydroxyapatite crystals in hydroxyapatite polycrystal
$\varvec{\varepsilon }_\text {gran}^\text {congl}$: Second-order strain tensor of granule material in bone-scaffold conglomerate
$\varvec{\varepsilon }_\text {gran}^\text {congl,dev}$: Second-order deviatoric strain tensor of granule material in bone-scaffold conglomerate
$\varvec{\varepsilon }_\text {gran}^\text {congl,vol}$: Second-order volumetric strain tensor of granule material in bone-scaffold conglomerate
$\varvec{\varepsilon }_\text {polyHA}^\text {gran}$: Second-order strain tensor of hydroxyapatite polycrystal in granule material
$\vartheta$: Angle defining the orientation of the spherical coordinate system (${\mathbf {e}}_r,{\mathbf {e}}_\vartheta ,{\mathbf {e}}_\varphi$)
$\mu _\text {gran}$: Shear modulus of granule material
$\mu _\text {HA}$: Shear modulus of hydroxyapatite crystals
$\mu _\text {congl}$: Shear modulus of bone-scaffold conglomerate
$\nu _\text {gran}$: Poisson’s ratio of granule material
$\nu _\text {HA}$: Poisson’s ratio of hydroxyapatite crystals
$\nu _\text {polyHA}$: Poisson’s ratio of hydroxyapatite polycrystal
$\varvec{\sigma }_\text {HA}^\text {polyHA}$: Second-order stress tensor of hydroxyapatite crystals in hydroxyapatite polycrystal
$$\sigma _{\text {HA},ij}^\text {polyHA}$$: Component of $$\varvec{\sigma }_\text {HA}^\text {polyHA}$$ ($$i,j=1,2,3$$)
$\sigma _{\text {HA}}^\text {ult,s}$: Shear strength of hydroxyapatite crystals
$\sigma _{\text {HA}}^\text {ult,t}$: Tensile strength of hydroxyapatite crystals
$\varvec{\sigma }_\text {gran}^\text {congl}$: second-order stress tensor of granule material in bone-scaffold conglomerate
$\varvec{\sigma }_\text {gran}^\text {congl,dev}$: Second-order deviatoric stress tensor of granule material in bone-scaffold conglomerate
$\varvec{\sigma }_\text {gran}^\text {congl,vol}$: Second-order volumetric stress tensor of granule material in bone-scaffold conglomerate
$$\sigma _{\text {gran},ij}^\text {congl}$$: Component of $$\varvec{\sigma }_\text {gran}^\text {congl}$$ ($$i,j=1,2,3$$)
$\varvec{\sigma }_\text {polyHA}^\text {gran}$: Second-order stress tensor of hydroxyapatite polycrystal in granule material
$$\sigma _{\text {polyHA},ij}^\text {gran}$$: Component of $$\varvec{\sigma }_\text {polyHA}^\text {gran}$$ ($$i,j=1,2,3$$)
$\varvec{\Sigma }_\text {congl}$: Second-order stress tensor of bone-scaffold conglomerate
$$\Sigma _{\text {congl},ij}$$: Component of $$\varvec{\Sigma }_\text {congl}$$ ($$i,j=1,2,3$$)
$\Sigma _{\text {congl},11}^\text {ult}$: Component in direction ${\mathbf {e}}_1$ of the second-order stress tensor of bone-scaffold conglomerate representing the ultimate loading
$\phi _\text {macro}^\text {congl}$: Volume fraction of macropores in bone-scaffold conglomerate
$\phi _\text {meso}^\text {gran}$: Volume fraction of mesopores in granule material
$\phi _\text {micro}^\text {polyHA}$: Volume fraction of micropores in hydroxyapatite polycrystal
$\varphi$: Angle defining the orientation of the spherical coordinate system (${\mathbf {e}}_r,{\mathbf {e}}_\vartheta ,{\mathbf {e}}_\varphi$)
$\psi$: Angle defining orientation of vector ${\mathbf {n}}$
